# 1331. High direct costs caused by Cryptococcal meningitis treated with Amphotericin B deoxycholate in a public hospital in Lima, Peru. 2021-2022

**DOI:** 10.1093/ofid/ofad500.1169

**Published:** 2023-11-27

**Authors:** Stalin Vilcarromero, Arturo Luque, Norka Luarte, Jimmy Mateo-Pacora, Fernando Mendo

**Affiliations:** Hospital Nacional Edgardo Rebagliati Martins. ESSALUD, LIMA, Lima, Peru; Hospital Edgardo Rebagliati Martins (HNERM), Lima, Lima, Peru; Hospital Edgardo Rebagliati Martins (HNERM), Lima, Lima, Peru; Hospital Edgardo Rebagliati Martins (HNERM), Lima, Lima, Peru; Hospital Edgardo Rebagliati Martins (HNERM), Lima, Lima, Peru

## Abstract

**Background:**

Cryptococcal meningitis (CM) is a severe disease caused by a ubiquitous fungus that affects both immunocompetent and immunosuppressed people. The treatment of choice includes liposomal amphotericin plus flucytosine; however, in Peru, both medications are not available. Hence, in Peru, CM is usually treated with Amphotericin B deoxycholate, despite adverse events that include longer hospital stays and a higher frequency of complications resulting in higher direct costs. The objective of this study is to evaluate the direct cost of CM in adults in the infectious disease service of the Edgardo Rebagliati Martins National Hospital in Lima, Peru. To do this, the clinical histories of adult patients hospitalized during the 2021 and 2022, and diagnosed by positive culture in the cerebrospinal fluid, were reviewed (Fig 1)

**Figure:**
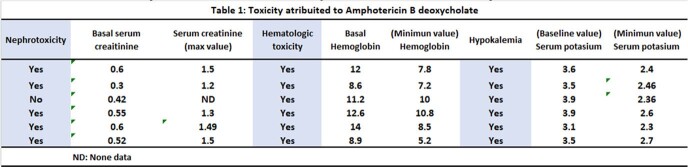

**Figure:**
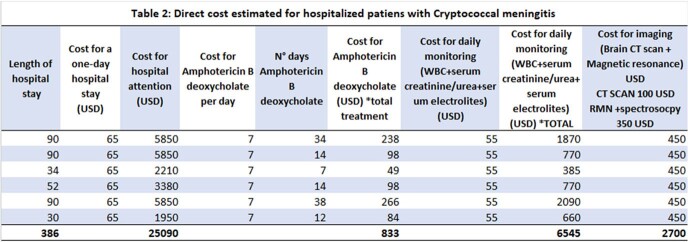

**Methods:**

Nine positive CM cases were identified; only six met the inclusion criteria. Daily hospitalization costs were estimated using published rates of IETSI-ESSALUD, a public employer-based health insurance. Costs for images and laboratory were based on private sector costs.

Positive culture in the cerebrospinal fluid for Cryptococcus sp

**Figure:**
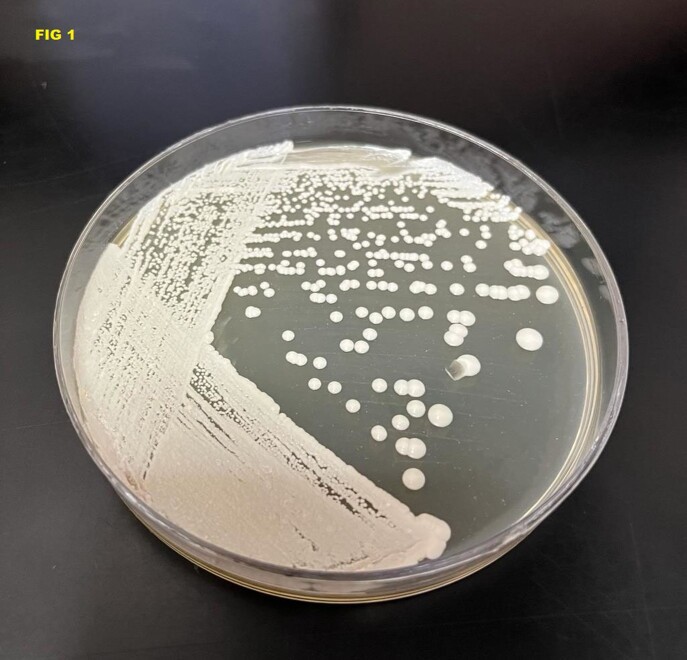

**Results:**

Three of the six cases were female and the mean age was 52.6 [39-61]. All six presented headache, nausea and vomiting. Three had HIV infection. All presented renal injury and hematological toxicity (Table 1). Three presented nosocomial infections. The average hospital stay was 66 days [30-90 days] with a total cost for all six patients of USD 35,168. The average direct cost per patient was USD 5,024 with the following distribution: average cost per hospital bed per person: USD 3,584.28; average cost of complete treatment with amphotericin b deoxycholate: USD 119; average cost for laboratory tests was USD 935; average cost for imaging was USD 385 USD (Table 2).

**Conclusion:**

The direct cost for CM treatment with amphotericin B deoxycholate is approximately USD 5,024; as a reference, the minimum monthly salary in Peru is USD 277. This high cost is not due to the drug, which is cheaper for the health system, but due to the long hospital stay and complications that occur. The small sample size is a great limitation; however, it partially reveals financial considerations that should be further and more thoroughly evaluated in the public health system in Peru.

**Disclosures:**

**All Authors**: No reported disclosures

